# Effects of the pandemic on women’s reproductive health protective attitudes: a Turkish sample

**DOI:** 10.1186/s12978-022-01412-y

**Published:** 2022-05-02

**Authors:** Derya Kaya Senol, Filiz Polat

**Affiliations:** Department of Midwifery, Faculty of Health Sciences, Osmaniye Korkut Ata University, Osmaniye, Turkey

**Keywords:** Women, COVID 19, Pandemic, Reproductive health protective attitudes, Midwife/nursing care

## Abstract

**Objectives:**

This descriptive, cross-sectional study was performed to examine the effects of the COVID-19 pandemic on women’s reproductive health protective attitudes.

**Methods:**

The study sample included 306 women and data were collected through a web-based, online questionnaire. The data were collected using the Personal Information Form, Determination of Married Women’s Reproductive Health Protective Attitudes Scale. Descriptive statistics, independent samples t-test, ANOVA test were used to assess the data.

**Results:**

The mean scores for Determination of Married Women's Reproductive Health Protective Attitudes Scale significantly differed in terms of education, employment status, income, health insurance and perceived health status (p < 0.05). A total of 69.3% of women had their first pregnancy at the agerange of 21–34 years, 17.6% of the women had four or more pregnancies, 55.6% of the women gave birth 1–3 times, 13.4% of the women gave birth at home and 57.8% of the women did not use modern family planning methods. A total of 23.2% of women experienced a problem with their reproductive organs during the pandemic, 70.6% of them did not present to a health center for their problems and 74.5% of these women did not present to a health center to avoid the risk of COVID-19 transmission. A total of 40.2% of women used the methods they already know at home to relieve their problems and 16.0% of the women used them edications previously prescribed by their doctors.

**Conclusion:**

The pandemic negatively affects there productive health of women. In the COVID-19 pandemic, health policies should be planned in accordance with the continuation of reproductive health and sexual health services.

## Plain language summary

Impairment of sexual and reproductive health (SRH) and inability to use sexual and reproductive rights are an important public health problem during pandemics. Reproductive healthcare services are limited to pregnancy and childbirth-related care during pandemics. This study was conducted to examine the effects of the pandemic on women’s protective attitudes towards reproductive health. In women in Turkey, education level, income status, health assurance, health perceptions, obstetric history and contraceptive methods are the factors that affect the reproductive health. The women presenting to health centers with reproductive health problems before a pandemic do not receive healthcare for their genital problems during the pandemic due to the risk of disease transmission.

## Introduction

A new type of coronavirus causing coronavirus disease (COVID-19) first appeared in China and rapidly spread throughout other countries [1]. Due to the severity of the disease and its international spread, the World Health Organization (WHO) declared the worldwide outbreak of COVID-19 on 31 January 2020 and a pandemic on 11 March 2020 [2–4]. The pandemic has created a global severe threat to public health and has been the third outbreak of a disease in humans due to coronavirus in the past 20 years [5].

Impairment of sexual and reproductive health (SRH) and inability to use sexual and reproductive rights are an important public health problem during pandemics. There is limited evidence about the clinical picture and outcomes created by COVID-19 in pregnancy and effects of the infection on SRH [4, 6]. Pandemics restrict access to healthcare especially preventive medicine and reproductive healthcare services and worsen the inequalities in the delivery of healthcare services. Although women benefit from more healthcare services at times other than pandemics, they experience more physical and mental problems on average every year compared to men [7]. Reproductive healthcare services are limited to pregnancy and childbirth-related care during pandemics [8]. However, access to all family planning services is equally important. In the framework of SRH, safe curettage, contraception, healthcare services for HIV/AIDS and other sexually transmitted diseases in addition to prenatal and postpartum screenings should not be disregarded [4].

Reproduction is essential for maintenance of the generations of all species. Human reproductive health has several aspects, i.e., physical, mental and social. Attempts to prevent and keep COVID-19 pandemic under control, the virus itself, drugs used for treatment, disinfectants, isolation conditions and precautions taken to protect public health have caused people to panic and experience some psychological problems [9]. In accordance with international and national healthcare policies during pandemic, health professionals started to work in the fields in which they did not specialize and especially the staff offering reproductive healthcare services were appointed to work in COVID-19 units. These changes have caused gaps in provision of the primary SRH services including prenatal and postpartum follow-up, safe curettage, contraception and healthcare for HIV-AIDS and sexually transmitted diseases. Therefore, the present study was performed to examine the effects of the pandemic on reproductive health protective attitudes of women.

## Methods

### Study design

This study has a descriptive, cross-sectional design and was performed to reveal the effects of the pandemic on women’s reproductive health protective attitudes between July and October in 2020.

### Sampling

The study population included married women aged 20–49 years. The region where the study was performed had a population of 268.647. According to data from the Turkish Population and Health Study (TPHS), 49.8% of the population were women in 2018, 48.9% of the female population were aged 20–49 years and 65.6% of this age group were married. The sample was calculated using the known sample calculation formula. Based on the confidence interval of 95% and the margin of error at 5%, the sample size representative of the study population was found to be a minimum of 382 [10]. In the study, 37 of the women were excluded because they did not meet the criteria and 39 of them filled in the data collection form incompletely.

### Inclusion criteria

The women aged 20–49 years, married, able to communicate, willing to fill in an online questionnaire and accepting to participate in the study were included in the study. Three hundred and eighty-two women were contacted. Out of 382 women, 76 not fulfilling the inclusion criteria were excluded, and the study was completed on 306 women satisfying the criteria.

### Exclusion criteria

Women who did not meet the research criteria and did not accept to participate in the study were excluded from the study.

### Data collection

Women were invited to the study through an online survey (Google Forms) link texted on WhatsApp. The data was collected by snowball sampling method. On the first page of the survey, the informed consent form with information on the purpose and procedure of the study was presented. Those who accept online participation marked the Confirmation link. After the consent has been issued the data collection form was displayed. Data Collection Form was composed of three sections. The first section had questions about sociodemographic and obstetric features, the second section had questions about knowledge and attitudes about COVID-19 and the third section had questions about reproductive health protective attitudes of married women.

### The first section of the questionnaire: sociodemographic, general health and obstetric features

The first section of the questionnaire was composed of questions about sociodemographic features including age, education, employment status, income, health insurance, smoking, general health status and presence of chronic diseases and obstetric features including pregnancy, labor, abortion and curettage, giving birth at home, the number of live children and contraceptives used.

### The second section of the questionnaire: Women’s knowledge and attitudes about COVID-19 and access to reproductive health services

The second section of the questionnaire was composed of questions about knowledge and attitudes of the women about COVID-19 like having the diagnosis of COVID-19, experiencing problems with the reproductive organs during the pandemic, presenting to a health center for the treatment of these problems, reasons for not going to a health center, what the women did when they avoided seeking help from a health center and effects of these problems on their daily life.

### The third section of the questionnaire: determination of Married Women’s Reproductive Health Protective Attitudes Scale (RHPAS)

Determination of Married Women’s Reproductive Health Protective Attitudes Scale (RHPAS) was developed and its validity and reliability were tested by Demirci (2004) [11]. It is a five-point self-report, easily understandable Likert scale composed of 39 items. The scale has five subscales; i.e., visits to a doctor for reproductive health related problems,protection against cancers of the reproductive organs and breasts, general health behaviors for reproductive health protection, protection against genital tract infections and prevention of unwanted pregnancies [11, 12]. At the beginning of the scale, information about how to fill in the scale is given. The women were asked to read each item in the scale and report how often they displayed the reproductive health protective attitudes and behaviors by marking one of the options provided: 1 corresponding to never, 2 very rarely, 3 sometimes, 4 mostly and 5 always. The items 5, 10, 16 and 28 were inversely scored. The total score for the scale ranges from 39 to 195. As the scores increase, reproductive health protective attitudes and behaviors are more favorable [11, 12]. Cronbach’s alpha for the scale was found to be 0.871 in the present study.

### Data analysis

Obtained data were analyzed with the Statistical Package Program for Social Sciences 21. Skewness and Kurtosis (± 1) were utilized to determine whether the data had a normal distribution. It was determined that all data were in accordance with the normal distribution and the analysis was made in this direction. Univariate analyses were used to examine the relations of the scores for RHPAS with descriptive variables. Student’s t-test was employed for comparisons of two groups and one-way variance analysis for comparisons of more than two groups. The results were evaluated by using the confidence interval of 95% and the significance level of p < 0.05.

### Ethical considerations

Ethical approval was obtained from the ethical committee of scientific research and publication at Osmaniye Korkut Ata University (Date: 22.06.2020, Approval Number: 2020/23/2). In addition, permission for scientific research about COVID-19 was obtained from the Turkish Ministry of Health (Form code: 2020-06-16T15_10_45). On the first page of the online questionnaire, an informed consent form was supplied. The participants were assured that participation in the study had a voluntary basis and that they could withdraw from the study when they wanted. They were informed that obtained data would be used for scientific purposes and published without reporting their names. Informed consent was obtained online from the participants in accordance with the Declaration of Helsinki.

## Results

The rate of women over 35 is 47.4%. A total of 59% of women had postgraduate education, 59.5% did not have a paid job, 24.2% had an income lower than their expenses, 20.9% did not have a health insurance, and 18.3% had a chronic disease. In the study, RHPAS points in high school graduates (145.7 ± 17.2) in the high school graduates (146.0 ± 18.2) in the employees (145.7 ± 18.2), in the health assurance (143.1 ± 20.7) in the health assurance (143.1 ± 20.7), in the health assurance (148.5 ± 21.7) was higher. The mean score for RHPAS was significantly different in terms of education, employment status, income, health insurance and perceived health status (p < 0.05) (Table 1).

**Table 1:** The comparisons of RHPAS scores in terms of sociodemographic features and general health status (n = 306)

	n (%)	RHPAS	Significance
		$\overline{\mathrm{X} }$ ± SS	
Age			
Younger than 25 years	88 (28.8)	140.9 ± 22.7	F = 0.083 p = 0.921
25–35 years	73 (33.9)	139.9 ± 19.4	
Older than 35 years	145 (47.4)	141.1 ± 19.8	
Education			
Illiterate	10 (3.3)	125.7 ± 13.6	*F = 7.276 p = 0.000
Primary education	66 (21.6)	132.8 ± 20.5	
High school	49 (16.0)	145.7 ± 17.2	
University and a higher education level	181 (59.2)	143.1 ± 20.6	
Employment			
Paid job	124 (40.5)	146.0 ± 18.2	**t = 3.805 p = 0.000
Unpaid job	182 (59.5)	137.1 ± 21.3	
Income			
Lower than expenses	78 (25.5)	131.5 ± 20.2	F = 11.795 p = 0.000
Equal to expenses	154 (50.3)	143.0 ± 20.4	
Higher than expenses	74 (24.2)	145.7 ± 18.2	
Health insurance			
Yes	242 (79.1)	143.1 ± 20.7	t = 3.911 p = 0.000
No	64 (20.9)	132.0 ± 17.2	
Smoking			
Smoker	45 (14.7)	140.1 ± 20.8	F = 2.866 p = 0.058
Ex-smoker	13 (4.2)	154.0 ± 19.6	
Nonsmoker	243(81.1)	140.3 ± 18.4	
Perceived health status			
Very good	16 (5.2)	148.5 ± 21.7	F = 7.184 p = 0.000
Good	162 (52.9)	144.6 ± 19.2	
Moderate	122 (39.9)	135.5 ± 20.8	
Poor	6 (2)	123.3 ± 14.1	
Presence of chronic diseases			
Yes	56 (18.3)	139.5 ± 21.6	t = − 0.519
No	250(81.7)	141.0 ± 20.3	p = 0.619

RHPAS: Determination of Married Women’s Reproductive Health Protective Attitudes Scale

*F = ANOVA test, **t = Independent Groups t-test

The mean age at menarche was 13.5 ± 1.6 years. A total of 69.3% of women had their first pregnancy at the age of 21–34 years, 17.6% had four or more pregnancies, 55.6% gave birth 1–3 times, and 57.8% did not use modern family planning methods. The mean score for RHPAS significantly differed with respect to age at the first pregnancy, number of childbirths, the number of live children, the number of giving births at home, using contraceptives and the type of the contraceptive used (p < 0.05) (Table 2).

**Table 2:** The comparisons of RHPAS scores in terms of obstetric features (n = 306)

	n (%)	RHPAS	Significance
		$\overline{\mathrm{X} }$ ± SS	
Age at the first pregnancy			
20 years or younger	76 (24.8)	133.2 ± 19.6	*F = 7.158 p = 0.001
21–34 years	212 (69.3)	143.0 ± 20.2	
35 years and older	18 (5.9)	145.5 ± 20.8	
The number of pregnancies			
None	96 (31.4)	140.4 ± 22.4	F = 1.438 p = 0.239
1–3	156 (51)	142.3 ± 19.3	
4 and higher	54 (17.6)	136.8 ± 20.4	
The number of childbirths			
None	97 (31.7)	140.6 ± 21.6	F = 4.451 p = 0.012
1–3	170 (55.6)	142.8 ± 20.0	
4 and higher	39 (12.7)	132.0 ± 18.0	
The number of live children			
None	95 (31.0)	140.6 ± 21.8	F = 4.827 p = 0.009
1–3	168 (54.9)	143.0 ± 20.1	
4 and higher	43 (14.1)	132.2 ± 17.5	
The number of abortions/curettages			
None	235 (76.8)	140.7 ± 20.6	F = 2.989 p = 0.055
1–2	61 (19.9)	138.6 ± 20.1	
3 and more	10 (3.3)	155.7 ± 17.4	
The number of childbirths at home			
None	265 (86.6)	141.7 ± 20.5	F = 4.448 p = 0.012
1–3	24 (7.8)	140.7 ± 17.4	
4 and higher	17 (5.6)	126.5 ± 20.0	
Using contraceptives			
Yes	129 (42.2)	143.9 ± 18.5	**t = 2.317
No	177 (57.8)	138.4 ± 21.6	p = 0.021
Types of contraceptives used			
Ria	38 (12.4)	136.6 ± 18.7	F = 3.472 p = 0.005
Condom	72 (23.5)	147.5 ± 18.4	
Tubal ligation	11 (3.6)	149.7 ± 24.2	
Withdrawal	149 (48.7)	137.3 ± 21.4	
Rhythm method	5 (1.6)	145.8 ± 7.1	
Pills	31 (10.1)	143.1 ± 19.2	
Age at Menarche		$\mathrm{Mean}:$ 13.5 ± 1.6	

RHPAS: Determination of Married Women's Reproductive Health Protective Attitudes Scale

*F = ANOVA test, **t = Independent Groups t-test

Twenty-seven percent of the women had a family member diagnosed with COVID-19, 23.2% experienced problems with the reproductive system during the pandemic, 70.6% did not present to a health center for the treatment of their problems and 74.5% of these women did not go to hospital for fear of contracting the infection. A total of 40.2% of women reported that they tried to find relief from their problem by using the methods they already used at home and 16.0% said they used the medications previously prescribed. The mean score for RHPAS was significantly different with regard to being diagnosed with COVID-19, the methods used to cope with the reproductive health problems before and after the pandemic, presenting to a health centers due to genital complaints and the reasons for not presenting to a health center (p < 0.05) (Table 3).

**Table 3:** The comparisons of RHPAS scores in terms of reproductive health behaviors during the pandemic (n = 306)

	n (%)	RHPAS	Significance
		$\overline{\mathrm{X} }$ ± SS	
Diagnosis of COVID-19			
A family member/I was diagnosed with COVID-19	20 (6.5)	139.9 ± 25.5	*F = 2.905 p = 0.056
A relative was diagnosed with COVID-19	83 (27.1)	136.8 ± 17.8	
No one was diagnosed with COVID-19	203 (66.3)	142.7 ± 21.4	
What did you use to do when you had a reproductive system problem before the pandemic?			
I used to go to the doctor	191 (62.4)	144.7 ± 20.7	F = 7.686 p = 0.000
I used to try finding relief by using a method I knew	33 (10.8)	138.7 ± 20.8	
I used to use medications prescribed before	11 (3.6)	133.0 ± 17.7	
I used to wait until it was relieved; if not, I used to g o to the doctor	71 (23.2)	132.1 ± 17.4	
Did you have a reproductive system problem during the pandemic?			
Yes	71 (23.2)	142.7 ± 18.8	**t = 0.914 p = 0.362
No	235 (76.8)	140.2 ± 21.0	
Did you go to a health center for your genital complaints?			
Yes	90 (29.4)	144.7 ± 19.1	t = 2.208 p = 0.028
No	216 (70.6)	139.1 ± 20.9	
Why didn’t you go to a health center?			
I don’t go to the doctor for my genital problems	35 (11.4)	131.9 ± 17.2	F = 3.667 p = 0.013
Due to the risk of COVID-19 transmission	228 (74.5)	141.3 ± 21.1	
Due to calls for staying at home	27 (8.8)	148.5 ± 17.4	
For fear of violating the social distance principles	16 (5.2)	138.5 ± 18.0	
What did you do about your reproductive system problems during the pandemic?			
I tried to find relief by using the methods I already knew	123 (40.2)	138.7 ± 19.2	F = 3.439 p = 0.009
I used the medications previously prescribed by my doctor	49 (16)	148.1 ± 19.4	
I did nothing and just waited until it was relieved	68 (22.2)	136.7 ± 21.8	
I used alternative methods like herbal tea and hot application	43 (14.1)	140.4 ± 20.5	
I took painkillers available at home	23 (7.5)	148.5 ± 22.0	
Did your genital complaints affect your daily life?			
Yes	138 (45.1)	140.1 ± 20.8	t = − 0.508 p = 0.612
No	168 (54.9)	141.3 ± 20.3	

RHPAS: Determination of Married Women’s Reproductive Health Protective Attitudes Scale

*F = ANOVA test, **t = Independent Groups t-test

The mean score on RHPAS was 140.7 ± 20.5 and the mean scores on its subscales were as follows 29.9 ± 6.6 on visits to a doctor for reproductive health related problems, 9.3 ± 4.2 on protection against cancers of the reproductive organs and breasts, 32.9 ± 7.0 on general health behaviors for reproductive health protection, 60.3 ± 7.4 on protection against genital tract infections and 11.7 ± 2.5 on prevention of unwanted pregnancies (Table 4).

**Table 4:** The mean scores and maximum and minimum scores for RHPAS and its subscales (n = 306)

	X ± SD	Min	Max
Determination of Married Women’s Reproductive Health Protective Attitudes Scale	140.7 ± 20.5	85	191
1. Visits to a doctor for reproductive health related problems	29.9 ± 6.6	11	40
2. Protection against cancers of the reproductive organs and breasts	9.3 ± 4.2	4	20
3. General health behaviors for reproductive health protection	32.9 ± 7.0	16	50
4. Protection against genital tract infections	60.3 ± 7.4	33	74
5. Prevention of unwanted pregnancies	11.7 ± 2.5	3	15

## Discussion

In the study, it was determined that education, employment status, income, health insurance and perceived health status, first gestational age, number of births, number of live children, number of births at home, use of birth control method and used birth control method affect reproductive health awareness levels of women. It was determined that women had problems with the reproductive system during the pandemic period, the majority of them did not apply to a health institution for the treatment of their problems due to the fear of infection, and about half of those who had problems tried to get rid of their problems with the methods they used at home or used previously prescribed drugs. It is seen that women cannot benefit from reproductive health services adequately during the pandemic period.

COVID-19 is a new threat to humans and there is little scientific evidence about its effects on SRH. Relevant research is limited to effects of COVID-19 on pregnancy and its transmission to the fetus [6]. There are many unresolved questions about the risk of COVID-19 transmission to the fetus during pregnancy, labor and breastfeeding, types of transmission and the relation of the disease with reproductive health problems.

Pandemics affect availability of routine healthcare to individuals and societies having a low income and not having regular physicals [13]. An income insufficient to meet needs, poverty and weakness are the factors having a negative effect on reproductive health and rights [14]. In the present study, the women having a low education level, an income lower than their expenses, a chronic disease and perceived poor health status and not having a paid job received a lower mean score for RHPAS. Kuşçu and Taşçı’s descriptive cross-sectional study, education, health insurance, contraceptive methods used and presence of reproductive health problem were effective in reproductive health protective attitudes [12]. More frequent follow-ups and more frequent visits to health centers would eliminate these factors and counseling offered by health professionals could have a positive effect on attitudes of the individuals to protection of their reproductive health.

The women aged 35 or over at their first pregnancy, having 1–3 pregnancies, labors and live children, not giving birth at home, using modern family planning methods had a higher mean score for RHPAS. According to data from the TPHS 2018, education and age groups of women have a positive relation with the number of pregnancies and labors. The women aged 35 years or older at their first pregnancy are high school graduates, university graduates or have a higher level of education. Age at first pregnancy is 23.6 years in women aged over 35 years and 22.5 years in women younger than 35 years [15]. In the present study, the women with a younger age at first marriage and first childbirth had a higher mean number of pregnancies and childbirths. In Turkish culture, women start to have an active sexual life when they get married and visit a doctor for obstetric or gynecological conditions. This may explain why they benefit more from reproductive healthcare services.

Previous pandemics were shown to result in decreased access to services for family planning, curettage, prenatal and postnatal care and gender based violence and mental healthcare and increased morbidity and mortality due to unwanted pregnancies, sexually transmitted infections and pregnancy related complications [16]. Diniz and Andrezzo’s descriptive cross-sectional study during the Zika virus disease outbreak in 2015, the rates of pregnancy-related complications and babies with inborn anomalies increased and debates about the women’s rights to have a safe curettage [17] and worries about reproduction and social justice which still continue appeared [18]. In the outbreak of Ebola virus disease in in West Africa between 2014 and 2016, the women offering care to the members of their families and frontline female healthcare professionals were exposed to higher risk of the infection [8].

Similar to other pandemics, COVID-19 has increased perceived risk in people and cause them to experience psychological problems [19, 20]. If psychological effects of pandemics are transformed into a chronic or traumatic dysfunction, then the quality of oocytes and reproduction outcomes are negatively affected. In a web-based cohort study in the USA and Canada, severe depression symptoms were found to have a relation with irregular menstrual cycles and decreased reproduction [21, 22]. It has been shown in the literature that previous pandemics have a negative impact on reproductive health. The rates of demands for curettage have been reported to increase in hospitals near Hunan, China, due to insufficient access to contraceptive methods or inability to know the effects of COVID-19 on pregnancy [4]. In a cross-sectional study conducted during the Zika epidemic in Puerto Rico, facilitating access to contraceptive methods was reported to prevent unwanted pregnancies and negative outcomes related to pregnancy and prenatal period [23]. Several studies have emphasized that during the outbreak of Ebola virus disease in Western Africa between 2014 and 2015, family planning services were disrupted and that the number of the women receiving prenatal care and giving birth in a health center decreased [24, 25]. In the descriptive cross-sectional study of with women from the United States of America, the women were found to change the contraceptive methods they used to postpone or prevent their pregnancy [26].

When effects of COVID-19-related stigmatization and discrimination and their impacts on patients with SRH problems and healthcare providers are taken into account, holistic healthcare services become of importance to meet clinical, epidemiological and psychosocial behavioral needs related to COVID-19, SRH and rights [4].

The percentage of the women presenting to a health center due to reproductive system problems (62.4%) was found to decrease during the pandemic (29.4%). Pandemics affect routine healthcare services. Spending long hours in the crowded waiting rooms to receive healthcare increases the risk of infection transmission [13]. In the current study, the most frequent cause of not presenting to a health center was the transmission of COVID-19.

In the study, the majority of women (n: 216, 70.6%) stated that they did not apply to a health institution for genital problems they experienced during the pandemic period. However, the reproductive health protective attitude scale scores were higher in individuals who had problems with their reproductive organs during the pandemic period. However, the average score was lower in women who did not apply to a health center despite having problems and who waiting until it was relieved. This result shows that women's awareness of protective attitudes towards reproductive health is high, but the pandemic has negatively affected service procurement. During pandemics, the changes in the healthcare system and appointment of the healthcare professionals in acute care units cause delays in other clinical services and healthcare professionals get infected and experience shortages of medical equipment [13]. These factors my prevent individuals from accessing and receiving healthcare services they need.

Due to the health policies modified by governments during pandemics, SRH services can be delayed and disregarded and women at reproductive age may experience some difficulties. It is expected that couples have a higher tendency and allocate more time for sexual relationship during lockdown. However, due to lockdown, couples cannot access contraceptives and healthcare services. These factors can cause millions of unwanted pregnancies, unsafe abortions and maternal deaths [27, 28]. In the present study, the mean score of the women for RHPAS was 140.7 ± 20.5. Total scores for the scale range from 39 to 195. Higher scores on the scale indicate positive protective behavior and attitudes about reproductive health [11]. The mean RHPAS score of the women in the present study showed that they had favorable attitudes to protection of reproductive health.

Family planning and public health centers should use their resources appropriately not to disrupt reproductive healthcare services during pandemics [29]. Disruption of primary SRH services or regarding them as unnecessary and a decreased usage of contraceptives can create an increase in unwanted pregnancies and a negative effect on women’s health especially in countries with a low or moderate economic status [30, 31].

### Limitations of the study

As the study was conducted in a state hospital in a city in East Anatolia in Turkey, The study is also restricted with the dates when it was performed, the data collection tool developed in accordance with the aim of the study and the responses given by the participants to the questions in the data collection tool.

## Conclusion

The women presenting to health centers with reproductive health problems before a pandemic do not receive healthcare for their genital problems during the pandemic due to the risk of disease transmission. Women use the drugs prescribed by the doctor for solutions to reproductive health problems, use alternative methods such as herbal tea, hot application, and take pain relievers. The reproductive health problems experienced by women affect their daily lives. While health policies are modified to satisfy increased demands for healthcare by patients with COVID-19 and to cope with the pandemic threat, it is important for countries to plan and maintain SRH services. Maintenance of reproductive health and family planning services will contribute to protection of women’s health and reduction of maternal deaths. The role of midwives and nurses is very important in the maintenance of reproductive health services. It is recommended to carry out studies covering more different geographies on reproductive health service disruption and women's reproductive health protective practices.

## Data Availability

The data that support the findings of this study are available from the corresponding author upon reasonable request.

## Abbreviations

RHPAS: Determination of Married Women’s Reproductive Health Protective Attitudes Scale
SRH: Sexual and reproductive health
WHO: World Health Organization
TPHS: Turkish Population and Health Study
